# Mevalonic acid exerts procoagulant effect by potentiating factor Xa

**DOI:** 10.1007/s13659-025-00564-1

**Published:** 2026-01-10

**Authors:** Liyuan Niu, Chuanfeng Liu, Shaoying Wang, Qikai Yin, Shiping Lin, Musan Yan, Wenshuo Li, Yuanjie Yin, Wei Wang, Wenjuan Yu, Xiaopeng Tang, Min Xue, Yuewei Wang

**Affiliations:** 1 Department of Vascular Surgery, The Affiliated Hospital of Qingdao University, Qingdao, 266003 Shandong China; 2 Department of Hematology, Affiliated Hospital of Qingdao University, Qingdao, 266003 China; 3 School of Basic Medicine, Qingdao University, Qingdao, 266071 Shandong China; 4 Department of Pathology, The Affiliated Hospital of Qingdao University, Qingdao, 266003 China; 5 Shandong Key Laboratory of Pathogenesis and Prevention of Brain Diseases, Qingdao, 266071 Shandong China

**Keywords:** Mevalonic acid, FXa, Inflammation, Thrombosis, Metabolic disorders

## Abstract

Thrombosis pathogenesis is closely linked to dysregulated lipid metabolism and inflammatory processes. However, the direct regulatory role of mevalonate pathway within the coagulation cascade is still not well understood. This study aimed to elucidate the regulatory effects of mevalonic acid (MVA) on the coagulation system. The effects of MVA on coagulation were measured by recalcification. Enzymatic kinetic analysis and natural substrate hydrolysis assays were performed to identify the coagulation target of MVA. Mice bleeding and thrombosis models were applied to evaluate the effects of MVA administration on hemostasis and thrombosis. Our current study reveals that MVA significantly accelerates plasma coagulation through potentiating the procoagulant activity of FXa, without influencing the platelet aggregation. Studies showed that MVA administration substantially shortened activated partial thromboplastin time, prothrombin time, and reduced bleeding time in both tail bleeding and saphenous vein injury models. Furthermore, using ferric chloride-induced thrombosis, deep vein thrombosis and cerebral infarction models, we observed that MVA markedly potentiated thrombus formation and stroke. Our findings establish for the first time that MVA directly regulates FXa procoagulant activity, while also suggesting potential crosstalk between lipid metabolic pathways and inflammatory signaling in coagulation modulation. These results provide novel mechanistic insights into coagulation abnormalities associated with metabolic disorders such as atherosclerosis and diabetes, highlighting the mevalonate pathway as a potential therapeutic target for thrombotic complications.

**Graphical Abstract:**

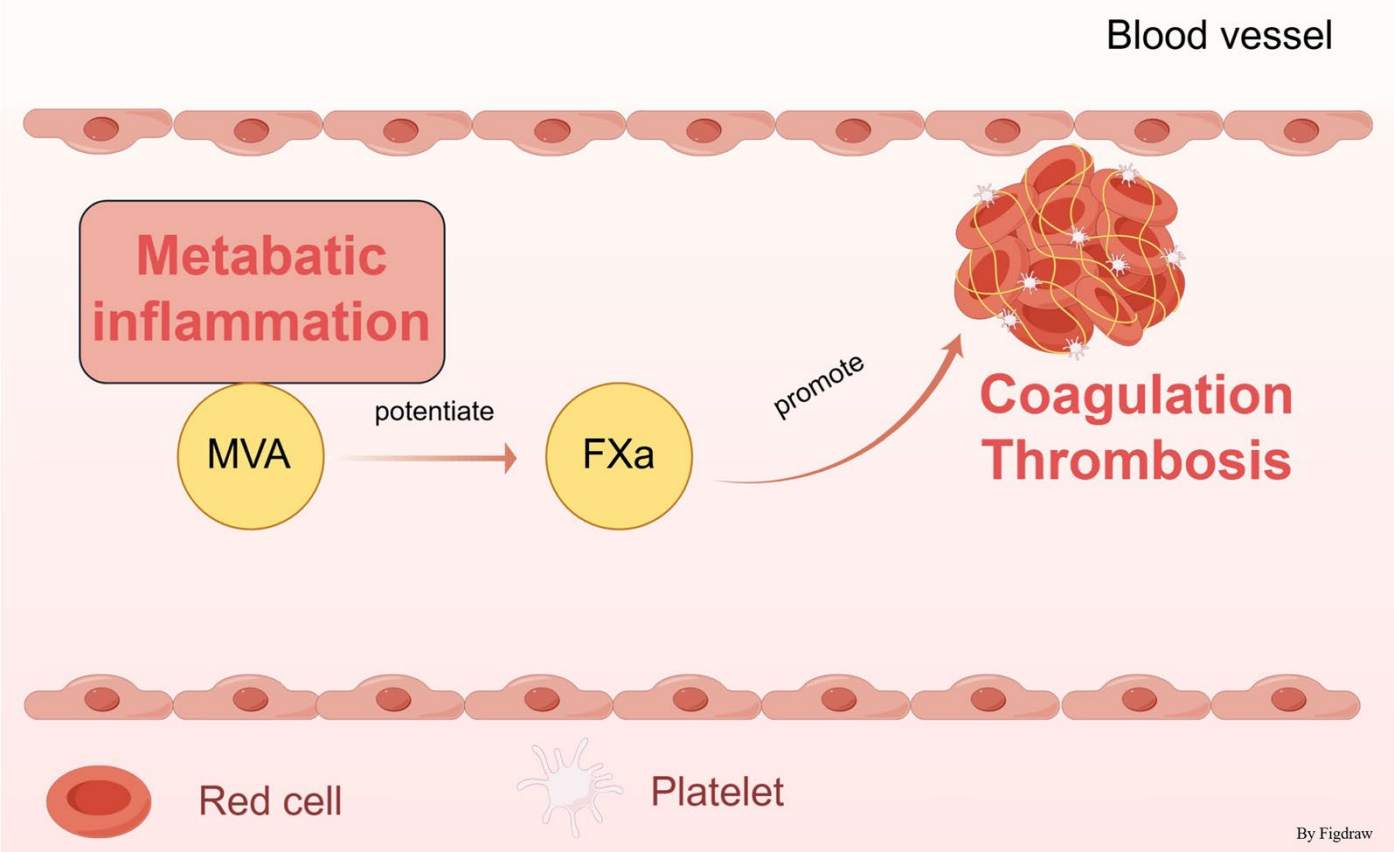

## Introduction

Metabolic inflammation, characterized by chronic low-grade inflammation driven by metabolic dysregulation, is linked to various metabolic disorders including hypertension, obesity, and dyslipidemia [1–4]. Lipid metabolism, particularly cholesterol homeostasis, critically influences thrombogenesis through multiple mechanisms: elevated LDL-C promotes a prothrombotic state by upregulating tissue factor expression and platelet aggregation via glycoprotein IIb/IIIa receptors, while HDL-C exerts antithrombotic effects by stimulating NO/prostacyclin production [5–9]. Additionally, LDL-C increases PAI-1 levels whereas HDL-C promotes t-PA release, collectively modulating fibrinolytic activity [10, 11]. These findings demonstrate that lipid metabolism regulates hemostatic balance through integrated effects on coagulation, fibrinolysis, and platelet function.

Mevalonic acid (MVA), a key intermediate metabolite in the cholesterol biosynthesis pathway, is a central component of the mevalonate pathway [12]. Metabolites derived from the mevalonate pathway, including isoprenoids, play a crucial role in regulating inflammatory signaling pathways, thereby influencing the initiation, progression, and resolution of inflammation through multiple mechanisms [13, 14]. The metabolites of the pathway regulate inflammation by modulating critical signaling cascades, including the NF-κB, mitogen-activated protein kinase (MAPK), and phosphatidylinositol 3-kinase (PI3K)/Akt pathways. These pathways are central to the inflammatory response, regulating the expression of pro-inflammatory mediators [15, 16]. Furthermore, mevalonate pathway plays a crucial role in the metabolism of LDL and HDL by regulating cholesterol synthesis and isoprenoid metabolites. In LDL metabolism, the mevalonate pathway modulates cholesterol synthesis through HMG-CoA reductase, influencing the expression of LDL receptors and the clearance of LDL [17]. Statins, which inhibit this pathway, effectively reduce LDL levels [18]. In HDL metabolism, the mevalonate pathway regulates the formation, maturation, and functionality of HDL through cholesterol synthesis and isoprenoid metabolites (farnesyl pyrophosphate), while also modulating HDL’s anti-inflammatory and antioxidant capacities by influencing inflammation and oxidative stress.

Although the current evidence suggests that mevalonate pathway may modulate inflammatory responses by modulating lipid synthesis in order to participate in the process of thrombosis, its direct regulatory role on the coagulation cascade remains unclear. Therefore, this study aims to elucidate the role of MVA in thrombosis by investigating its regulatory effects on the coagulation pathway. In the present study, we elucidated the direct effect of MVA on the coagulation cascade and demonstrated that MVA induces a hypercoagulable state by potentiating FXa activity, thereby promoting thrombus formation.

## Method

### Animals and ethics statement

All animal experiments performed in this study were carried out in compliance with the guidelines outlined in the Guide for the Care and Use of Laboratory Animals and were approved by the Animal Care and Use Committee of Qingdao University (QDU-AEC-2025559) and Qingdao Central Hospital of University of Health and Rehabilitation Sciences (KY202319301). C57BL/C mice (8 weeks old) were from Vitalriver Experiment Animal Company (Beijing, China).

### Effects of MVA on coagulation and platelet aggregation

To prepare plasma samples, whole blood collected from healthy donors was anticoagulated with 0.13 M sodium citrate at a 1:9 ratio (citrate: blood). The anticoagulated blood was then subjected to centrifugation at 3000 rpm for 30 min using a high-speed refrigerated centrifuge. For the evaluation of MVA-induced calcification, mevalonic acid lithium salt (HY-113071A, MCE, USA) was prepared with 40 μl of plasma. This mixture was maintained at 37 °C for 10 min before the addition of 50 μl pre-warmed 25 mM CaCl_2_ (37 °C). The coagulation process was monitored by measuring optical density at 650 nm using a microplate reader (Readmax 1500, Shanpu, Shanghai, China). The coagulation time was determined as the time point when the absorbance reached 50% of its maximal value, based on the recorded kinetic curves.

Blood samples were collected using sodium citrate (1:9) as an anticoagulant. Platelet-rich plasma (PRP) was immediately isolated through centrifugation at 150 g for 10 min under room temperature conditions. The PRP was subsequently treated with MVA (70 nM) and incubated at 37 °C for 5 min. Platelet aggregation was induced by either collagen (2 μg/ml, AG005K-RUO, Hyphen BioMed, France), thrombin (1 μg/ml, HT 1002a, Enzyme Research Laboratories, USA), arachidonic acid (2 μg/ml, HY-109590, MCE, USA) or ADP (6 μΜ, A2754, SIGMA, USA), with the aggregation process monitored using a platelet aggregometer (AG400, Techlink Biomedical, China). The maximum aggregation rate was determined as the primary outcome measure.

### Effects of MVA on coagulation factors

MVA effects on coagulation-related enzymes and inhibitors (e.g., kallikrein, FXIIa, FXIa, FXa, thrombin, antithrombin, plasmin, antiplasmin) were evaluated using the corresponding chromogenic substrates. The enzyme was incubated with MVA in 60 μl of 50 mM Tris-HCl buffer (pH 7.4) for 5 min. Subsequently, 0.2 mM of the corresponding chromogenic substrate was added to the reaction mixture. The absorbance at 405 nm was measured immediately, and the kinetic curve was recorded using an enzyme labeler (Readmax 1500, Shanpu, China). The relative enzyme activity was calculated based on the substrate hydrolysis rate. The chromogenic substrate S-2238 (0.2 mM, Chromogenix AB, Sweden) was incubated with either human α-thrombin (20 nM) or human plasmin (20 nM, HPlasmin, Enzyme Research Laboratories, USA). The chromogenic substrate S-2302 (0.2 mM, Chromogenix AB, Sweden) was incubated with human α-FXIIa (50 nM, HFXIIa 1212a, Enzyme Research Laboratories, USA) and kallikrein (20 nM, HPKa 1303, Enzyme Research Laboratories, USA). The chromogenic substrate S-2222 (0.2 mM, Chromogenix AB, Sweden) was used for FXa (30 nM, HFXa 1011, Enzyme Research Laboratories, USA). Additionally, the chromogenic substrate S-2366 (0.2 mM, Chromogenix AB, Sweden) was incubated with FXIa (20 nM, HFVIIa, Enzyme Research Laboratories, USA).

The effect of MVA on the catalytic activity of FXa in relation to its natural substrate, prothrombin, was similarly evaluated. FXa (0.1 μg) was incubated with prothrombin (1 μg) in TRIS-HCl buffer containing MVA (70 or 210 nM) for 35 min at 37 °C. The enzymatic activity of FXa against prothrombin in the presence of MVA was assessed by SDS-PAGE, followed by staining with Coomassie brilliant blue. A separate SDS-PAGE gel was transferred onto polyvinylidene difluoride (PVDF) membranes, which were then blocked with 5% bovine serum albumin (BSA) in TBST buffer for 2 h at ambient temperature. Following three washes with TBST buffer, the membranes were incubated with primary anti-prothrombin antibody (dilution 1:200, MK82058S, Abmart, China) at 4 °C overnight. After an additional three TBST washes, incubation with secondary antibody was performed at room temperature for 1 h. The membranes were subsequently subjected to another round of TBST washing before being developed using an enhanced chemiluminescence kit (PA112, Tiangen, China) on an ImageQuant LAS 4000 mini system (GE Healthcare, USA).

### Flow cytometry

Flow cytometry was employed to assess αIIbβ3 activation, P-selectin expression, and phosphatidylserine exposure, utilizing FITC-conjugated anti-P-selectin antibody (561923, BD Biosciences, USA), FITC-conjugated PAC-1 (MA5-28564, Invitrogen, USA), and FITC Annexin V (640905, BioLegend, USA), respectively. Following stimulation, washed platelets were incubated for 30 min at 37 ℃ under dark conditions prior to analysis on a Beckman Coulter MoFlo XDP flow cytometer. Data acquisition included 10,000 events, with subsequent analysis performed using FlowJo software (version 10.8.1).

### Activated partial thromboplastin time (APTT) and prothrombin time (PT) assays

MVA was administered via tail vein injection for 5 min, after which blood samples were collected. Plasma was prepared using the previously described method. For the APTT assay, 100 μl of plasma was combined with 100 μl of APTT reagent (R41056-50T, Yuanye, China) and pre-incubated at 37 °C for 5 min. Subsequently, 100 μl of 25 mM calcium chloride solution was added, and the mixture was incubated at 37 °C to measure the clotting time. For the PT assay, 75 μl of plasma and PT reagent were separately preheated at 37 °C for 5 min, then mixed and immediately incubated at 37 °C to determine the clotting time. Furthermore, the effect of MVA at concentrations of 70 nM or 210 nM on APTT and PT was also detected in vitro.

### Bleeding time measurement

The mice tail-bleeding assay was performed following established methodologies described in our previous publications [19]. To assess bleeding time, 5 min after MVA injection, a 4-mm segment was precisely excised from the tip of each mouse tail and immediately immersed in 10 mL of pre-warmed saline solution maintained at 37 °C. Cessation of bleeding for 2 min was employed as the endpoint of the experiment. To prevent death of the mice, hemorrhage was observed for no more than 20 min.

### Saphenous vein bleeding model

As previous described in our study [19, 20], saphenous vein bleeding model was employed to evaluate the effects of MVA on hemorrhage. Following exposure of the saphenous vein in mice, the time required for spontaneous hemostasis within a 30-min period was recorded. After each hemostatic event, the blood clot was disrupted, and the duration from the initiation of bleeding to cessation was documented.

### FeCl_3_-induced carotid artery thrombosis

As described in our previously published protocols [21], the FeCl_3_-induced thrombosis model was utilized to evaluate the regulatory effects of MVA on thrombus formation. Thrombosis in the carotid artery induced by 10% FeCl_3_ was monitored until complete occlusion using laser speckle perfusion imaging (RFLSI III, RWD Life Science, China) after mice were anesthetized using isoflurane.

### Mice deep vein thrombosis model

The DVT model was conducted according to the standard methods outlined in our previous publications [22]. Mice were anesthetized, followed by ligation of the venous lateral and dorsal branches of the inferior vena cava (IVC) as well as the infrarenal IVC to induce stasis thrombosis. After 5 h, the mice were euthanized under anesthesia, and the thrombi were harvested for measurement of thrombus length and weight.

Pathological sections were prepared using frozen sectioning. Thrombus specimens were excised and fixed in 2% paraformaldehyde for 24 h. Subsequently, the specimens were transferred into a 30% sucrose solution for dehydration over a period of 48 h, with the sucrose solution being replaced once during this process. Upon completion of dehydration, the thrombus specimens were embedded in an embedding agent and sectioned to a thickness of 8–10 μm using a frozen sectioning machine. Hematoxylin and eosin (HE) staining was then performed on the sections.

### Mice stroke model

As previous described in our study [21], a middle cerebral artery occlusion (MCAO) model was employed to assess the effects of MVA on the occurrence of cerebral infarction. Following anesthesia, the common carotid artery (CCA), internal carotid artery (ICA), and external carotid artery (ECA) were exposed in mice. A silicon-coated nylon suture (6023910PK10, Doccol, Sharon, MA) was inserted through the bifurcation of the CCA into the ICA and advanced to the origin of the right middle cerebral artery (MCA) to induce occlusion. After 1 h, the suture was withdrawn to allow reperfusion. Twenty-four hours post-reperfusion, the mice were euthanized, and brain tissues were harvested. Coronal sections (2 mm thick) were prepared using a Rodent Brain Matrix (Harvard Apparatus, Holliston, MA). The sections were stained with 2% 2,3,5-triphenyltetrazolium chloride (TTC, Sigma, St. Louis, MO) to assess the ischemic areas. The Bederson test assesses hemiplegia and neurological deficits by observing forelimb symmetry and trunk posture when the animal is held by the tail. The scoring criteria are as follows: Grade 0 (bilateral forelimbs symmetric with no abnormalities); Grade 1 (mild flexion of the affected forelimb without trunk deviation); Grade 2 (marked curling of the affected forelimb with trunk rotation toward the affected side); Grade 3 (complete curling of the affected forelimb with severe trunk tilting or rolling). The Grip test (grip strength test) quantitatively evaluates limb muscle strength by measuring grip force using a dynamometer, with qualitative references: Grade 0 (inability to grasp); Grade 1 (brief touch only, grip strength < 25% of normal); Grade 2 (partial grasp with rapid release, grip strength 25–50% of normal); Grade 3 (stable grasp with weak force, grip strength 50–75% of normal); Grade 4 (grip strength > 75% of normal).

### Statistical analysis

The results of independent experiments are presented as mean ± standard deviation (mean ± SD). A two-tailed test was employed for all statistical analyses, with a confidence interval (CI) of 95%. The normal distribution of data was assessed using the Kolmogorov–Smirnov test (K-S test). Subsequently, a one-way analysis of variance (one-way ANOVA) was conducted, followed by post hoc Dunnett’s test for multiple comparisons. Between-group comparisons were performed using unpaired t-tests, while non-parametric data were analyzed with the Mann–Whitney U test. All data were processed using Prism 9.5 (GraphPad Software) and SPSS (SPSS Inc., USA). A *p*-value of less than 0.05 (*p* < 0.05) was considered statistically significant.

## Result

### MVA promotes blood coagulation but does not affect platelet aggregation

**Fig. 1:**
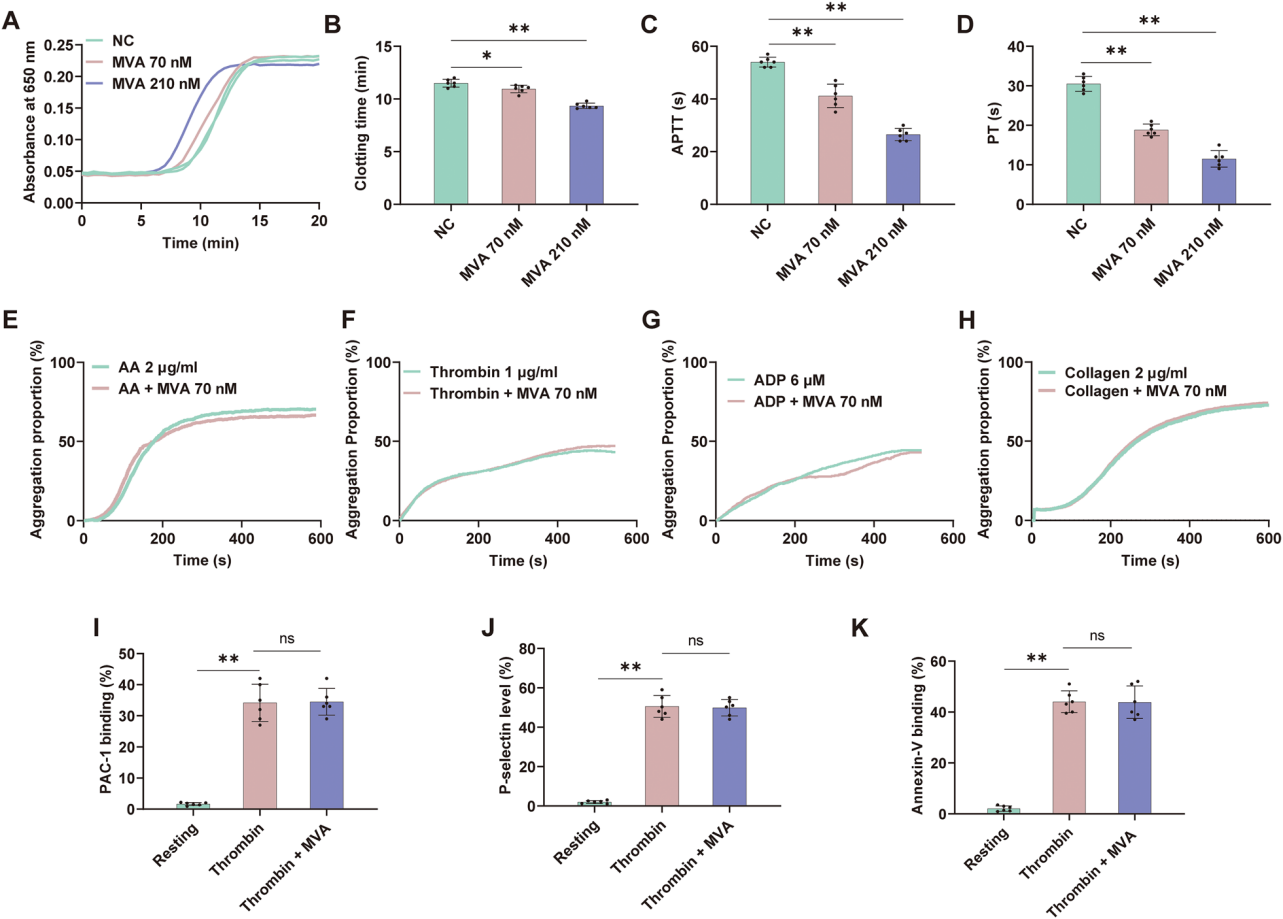
Mevalonic acid potentiates FXa but shows no effect on platelet aggregation. **A**, **B** Plasma recalcification time was shortened by Mevalonic acid (MVA). Effects of MVA (70 nM or 210 nM) on APTT (**C**) and PT (**D**) in vitro*.* MVA showed no effect on arachidonic acid (2 μg/ml) (**E**), thrombin (1 μg/ml) (**F**), ADP (6 μM) (**G**), collagen (2 μg/ml) (**H**). MVA exerted no significant influence on platelet activation markers, including αIIbβ3 integrin activation (**I**), P-selectin expression (**J**), and phosphatidylserine exposure (**K**). Data represent mean ± SD of 6 independent experiments, **p < 0.01, *p < 0.05 by one-way ANOVA with Dunnett’s post hoc test (**B**–**D**) or two-tailed unpaired Student’s t test (**I**–**K**). *NS* no significance

Building upon the crucial regulatory function of metabolic inflammation within the coagulation pathway, our objective is to assess the phenotypic effects of MVA, a vital intermediate in cholesterol metabolism, on coagulation function. This evaluation aims to establish a phenotypic foundation for elucidating the pathological mechanism by which metabolic abnormalities directly interfere with the coagulation cascade. We initially assessed the impact of MVA on coagulation and platelet through recalcification experiments and platelet aggregation assays. As illustrated in Fig. 1A, MVA dose-dependently reduced blood clotting time in the recalcification assay. At concentrations of 70 and 210 nM, MVA reduced the clotting time from 11.5 to 10.9 min and 9.4 min, respectively (Fig. 1B). In addition, MVA exerted a significant reduction in APTT and PT compared to the control group in vitro (Fig. 1C, D). To assess whether MVA affects platelet aggregation, we performed a platelet aggregation assay. MVA had no significant effect on platelet aggregation induced by arachidonic acid, ADP, collagen or thrombin with relative weak platelet aggregation (Fig. 1E–H). As illustrated in Fig. 1I–K, MVA exerted no significant effect on platelet activation markers, including αIIbβ3 integrin activation, P-selectin expression, and phosphatidylserine exposure. These findings suggest that MVA may exert a procoagulant effect, potentially through promoting the activation of the coagulation cascade rather than platelet aggregation.

### MVA potentiates FXa

**Fig. 2:**
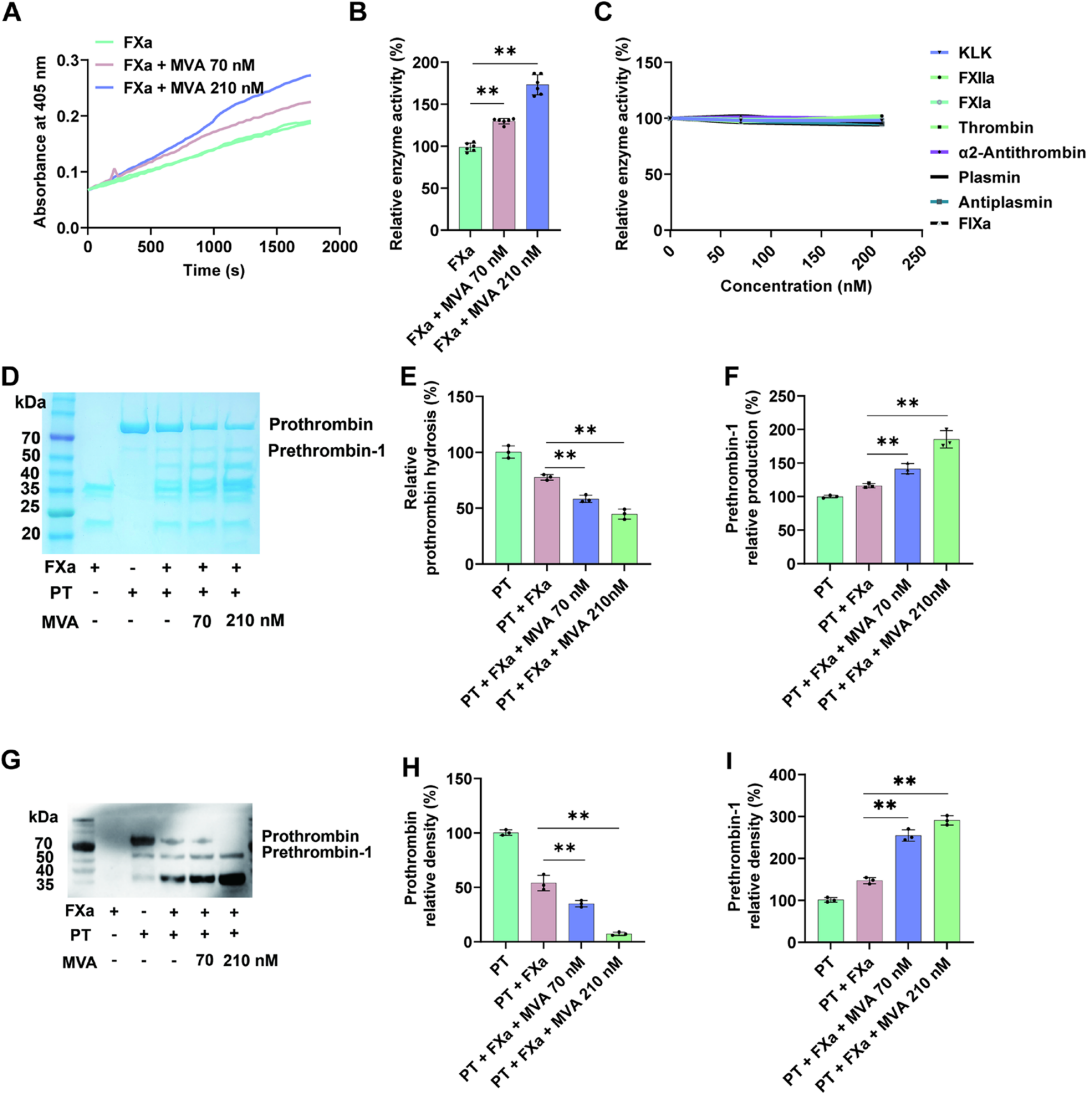
Mevalonic acid increases the FXa catalytic activity. **A**, **B** Potentiating effects of Mevalonic acid (MVA) on FXa. **C** MVA showed no influence on Kallikrein, FXIIa, FXIa, plasmin, anti-plasmin, thrombin, or anti-thrombin. **D** Representative SDS-PAGE analysis of prothrombin hydrolysis by FXa, and quantification of prothrombin and prethrombin 1 shown in **E**, **F**. **G** Prothrombin hydrolysis was analyzed by western blot, and corresponding quantification of prothrombin and prethrombin 1 shown in **H**, **I**. Lane 1: 0.1 μg FXa; Lane 2: 1 μg prothrombin; lane 3, 4, 5: 1 μg prothrombin + 0.1 μg FXa + 0, 70, 210 nM MVA. Data represent mean ± SD of 3 or 6 independent experiments, **p < 0.01, by one-way ANOVA with Dunnett’s post hoc test

To elucidate the underlying mechanisms of MVA-mediated coagulation enhancement, we conducted kinetic assays to systematically investigate the effects of MVA on both coagulation factors and their corresponding inhibitors. Result showed MVA potentiate the activity of FXa (Fig. 2A). Furthermore, a concentration of 70 nM MVA increased the efficiency of FXa hydrolysis of the synthetic substrate by 1.3-fold, while a concentration of 210 nM MVA further enhanced the efficiency to 1.7-fold (Fig. 2B). Notably, MVA showed no significant effects on KLK, FXIIa, FXIa, plasmin, anti-plasmin, thrombin, or anti-thrombin (Fig. 2C). Furthermore, MVA also potentiated the enzymatic activity of FXa towards its natural substrates: prothrombin [23]. As shown in the Fig. 2D–I, MVA at concentrations of 70 nM and 210 nM increased the prothrombin hydrolysis rate by ~1.6-fold and ~2.2-fold, and elevated production of prethrombin 1 by ~1.5-fold and ~1.8-fold, respectively. Collectively, these studies indicate that MVA promotes blood coagulation in a dose-dependent manner by enhancing FXa activity.

### MVA promotes coagulation and hemostasis

**Fig. 3:**
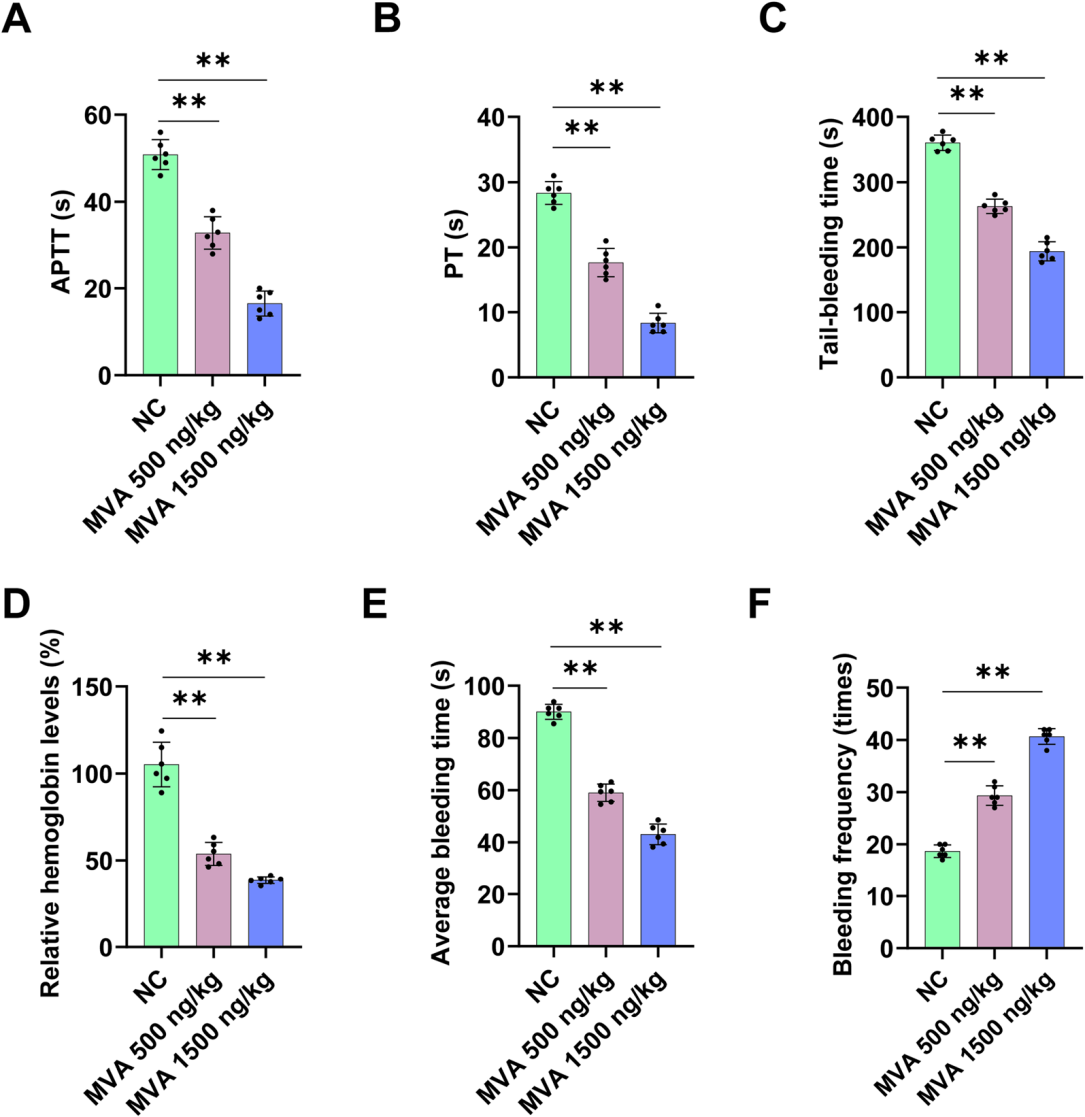
Mevalonic acid accelerates coagulation and induces hemostasis. Effects of intravenous injection of mice MVA (500 ng/kg or 1500 ng/kg) on APTT (**A**) and PT (**B**) ex vivo, mice tail-bleeding time (**C**) and Hemoglobin levels (**D**), mice saphenous vein bleeding time (**E**) and bleeding times (**F**). Data represent mean ± SD of 3–6 independent experiments, **p < 0.01, by one-way ANOVA with Dunnett’s post hoc test

To further evaluate the role of MVA in coagulation, experiments were performed to assess the effect of MVA administration on APTT, PT, tail-bleeding time and saphenous vein bleeding time. MVA (500 or 1500 ng/kg) was intravenously injected to C57BL/C mice through the tail vein, followed by blood collection for APTT and PT analysis. MVA treatment led to a significant reduction in APTT and PT compared to the control group ex vivo (Fig. 3A, B). Subsequently, tail-bleeding and saphenous vein bleeding assays were conducted under the same dosing protocol. The role of MVA in hemostasis was further investigated using mice tail-bleeding and saphenous vein bleeding models. As demonstrated in Fig. 3C, D, following the administration of 500 and 1500 ng/kg MVA, the tail-bleeding time in mice decreased, while the hemoglobin concentration was reduced to 54 and 38%, respectively. Significantly shorten saphenous vein bleeding time was found with MVA treatment, while the bleeding times were increased (Fig. 3E, F). These findings collectively demonstrate that MVA plays a crucial role in coagulation and hemostasis.

### MVA aggravates thrombosis

**Fig. 4:**
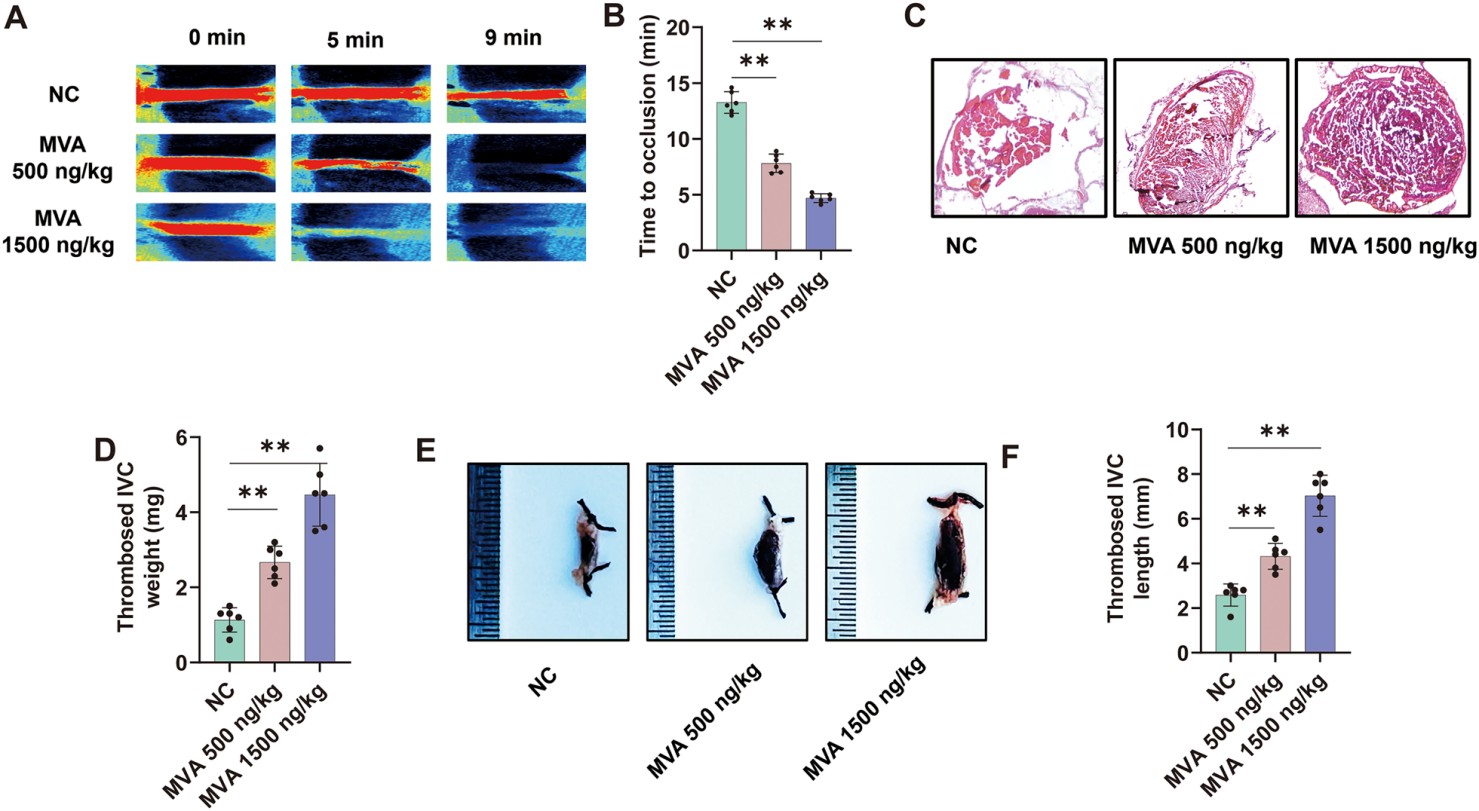
Mevalonic acid increases thrombosis risk. Mice were administered injections of either 500 ng/kg or 1500 ng/kg Mevalonic acid (MVA). **A** Carotid blood flow in FeCl_3_-treated mice was observed using laser speckle perfusion imaging to quantify changes in blood flow. **B** Time to occlusion was calculated in these mice group. Mice were subjected to the inferior vena cava IVC stasis model for 5 h to evaluate venous thrombosis formation, with subsequent pathological assessment by hematoxylin and eosin (HE) staining (**C**) and thrombus weight was quantitatively measured (**D**). **E**, **F** Thrombus length was measured. Data represent mean ± SD of 3–6 independent experiments, **p < 0.01, by one-way ANOVA with Dunnett’s post hoc test

We have previously demonstrated that MVA can enhance blood coagulation by increasing FXa activity, thereby reducing bleeding time. However, whether MVA can promote thrombus formation remains unclear. To further investigate the impact of MVA on thrombosis, FeCl_3_-induced carotid artery thrombosis, inferior vena cava (IVC) thrombosis models were established in mice. The drug administration protocol followed the previously established method. As shown in Fig. 4A, B, intravenous administration of MVA significantly reduced clotting time in mice subjected to 12% FeCl_3_-induced thrombosis, The clotting times for the 500 and 1500 ng/kg MVA injection groups were 7.8 and 4.7 min, respectively, whereas about 13.3 min in the control group. Furthermore, MVA treatment markedly increased both thrombus weight (Fig. 4C, D) and length (Fig. 4E, F). These results suggest that MVA plays a crucial role in regulating thrombus pathogenesis.

### MVA increases the risk of cerebral infarction

**Fig. 5:**
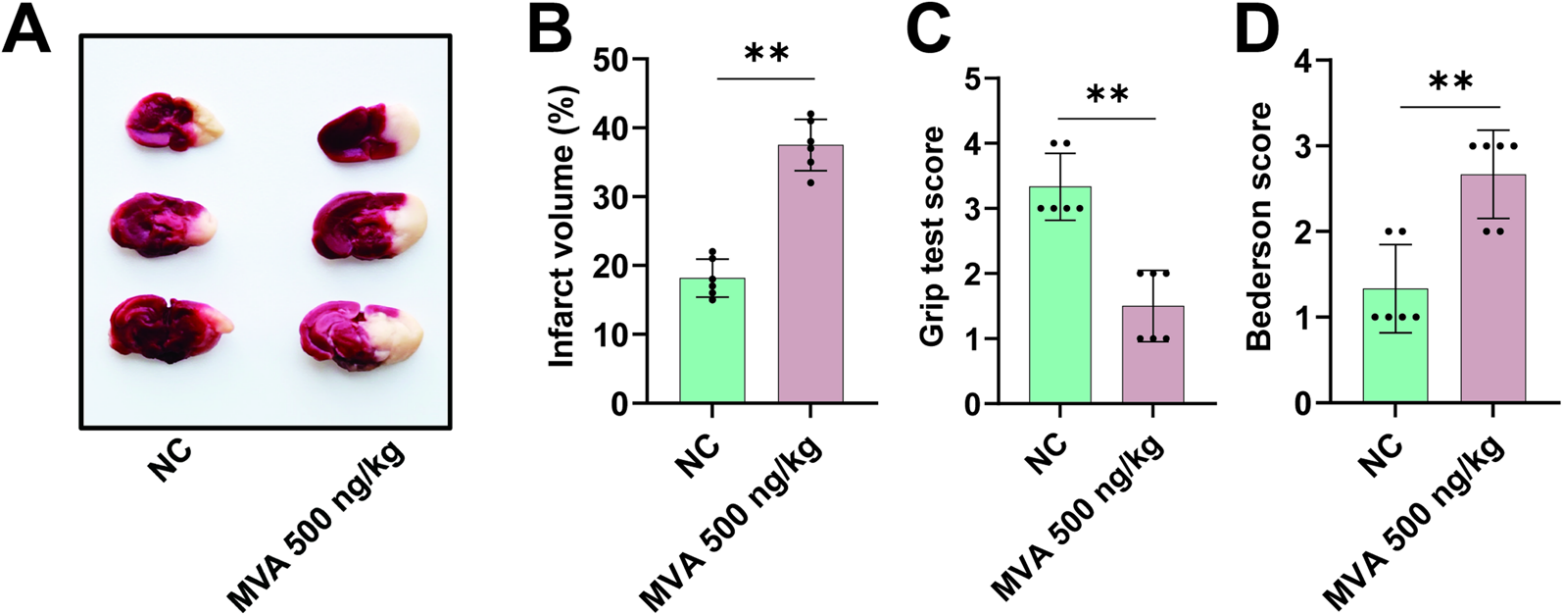
Mevalonic acid promotes cerebral infarction. Mice were administered injections of either 500 ng/kg Mevalonic acid (MVA). On 24 h after transient middle cerebral artery occlusion (tMCAO), typical images of coronal brain sections stained with 2,3,5-triphenyltetrazolium chloride (TTC) (**A**) and quantitative analysis of stained area (**B**). Bederson score (**C**) and grip test score (**D**) were also measured. Data represent mean ± SD of 3 independent experiments, **p < 0.01, by two-tailed unpaired Student’s *t* test

Hypercoagulability significantly contributes to cerebral infarction pathogenesis by promoting thromboembolic events [24, 25]. The prothrombotic milieu also exacerbates post-ischemic injury by amplifying microvascular thrombosis and compromising collateral circulation. To evaluate the potential association between MVA, coagulation, and cerebral infarction risk, an ischemic stroke model was employed. As shown in Fig. 5, MVA led to a significant increase in infarct volume (Fig. 5A, B) and severe functional outcomes, as evidenced by elevated Bederson scores and reduced grip strength scores (Fig. 5C, D).

## Discussion

Metabolic inflammation plays a significant role in promoting the development and progression of cardiovascular diseases (CVD) [26–28] along with hypercoagulable states. Chronic low-grade inflammation promotes atherosclerotic plaque progression by facilitating foam cell formation, endothelial dysfunction and inducing gut microbiota dysbiosis, thereby increasing the risk of cardiovascular events and thromboembolic complications [29–32]. As a critical metabolic pathway for lipid synthesis, the mevalonate pathway exerts its effects on inflammatory states both indirectly, by modulating cholesterol synthesis, and directly, by regulating the activity of inflammatory factors and the activation of inflammatory signaling cascades [33, 34]. These observations suggest that the mevalonate pathway contributes to the regulation of coagulation balance via indirect mechanisms. This study found that MVA significantly enhances the procoagulant activity of FXa, which can reduce bleeding time and increase the risk of thrombosis and cerebral infarction, providing new insights into the regulatory mechanisms of coagulation. The existence of a direct role for MVA acting on coagulation factors in addition to the indirect pathway involved in coagulation through inflammation was discovered.

Given the pivotal role of the mevalonate pathway in cholesterol biosynthesis and cardiovascular disease pathogenesis [35, 36], therapeutic agents targeting this metabolic pathway are under active development [37, 38]. Statins competitively inhibit HMG-CoA reductase [35, 39], the rate-limiting enzyme of the mevalonate pathway, thereby blocking the conversion of HMG-CoA to mevalonate. This inhibition reduces the production of downstream metabolites, including cholesterol and isoprenoids (FPP and GGPP). Additionally, statins upregulate LDL receptor expression through SREBP-2 modulation [40], enhancing LDL clearance [41]. However, current therapeutic strategies remain primarily focused on indirect regulators of coagulation balance, such as lipid levels, inflammation, and oxidative stress [42]. In this study, we have elucidated the direct regulatory effect of MVA, a critical intermediate in lipid metabolism, on coagulation factor FXa. Specifically, MVA directly potentiates FXa, thereby promoting coagulation and thrombus formation. This discovery provides a novel direction for identifying new therapeutic targets and developing innovative drugs.

While our current investigation has yielded significant mechanistic insights, the scope of findings remains constrained. Specifically, we have unequivocally established that the mevalonate pathway enhances FXa activity—a pivotal finding that nevertheless represents only the initial elucidation of the intricate crosstalk between metabolic regulation and coagulation cascades. To comprehensively delineate this relationship, we have designed a multifaceted experimental strategy: First, we will implement site-directed mutagenesis of FXa. Systematic introduction of missense mutations at predicted functional domains will allow precise mapping of residue-specific contributions to mevalonate pathway interactions. This mutagenesis approach will identify critical amino acid residues governing the molecular recognition events. Subsequently, we will perform rigorous computational analyses through molecular docking simulations. These in silico experiments will generate atomic-resolution structural models of FXa-MVA complexes, enabling quantitative characterization of interaction thermodynamics (binding free energies) and stereochemical complementarity (binding poses). Furthermore, we will develop targeted peptide-based interference assays. Rational design of high-affinity inhibitory peptides that competitively occupy the identified binding interfaces will provide orthogonal validation of the functional interaction sites through both biochemical and cellular approaches.

In conclusion, this study is the first to reveal the direct regulatory effect of MVA on the procoagulant activity of FXa, providing a new perspective for understanding coagulation regulation mechanisms. This discovery not only expands our understanding of the biological functions of MVA but also offers potential targets for the development of novel anticoagulant drugs.

## Data Availability

The raw data supporting the conclusions of this article will be made available by the authors on request.
